# Why do nursing students leave bachelor program? Findings from a qualitative descriptive study

**DOI:** 10.1186/s12912-022-00851-z

**Published:** 2022-03-29

**Authors:** Federica Canzan, Luisa Saiani, Elisabetta Mezzalira, Elisabetta Allegrini, Arianna Caliaro, Elisa Ambrosi

**Affiliations:** 1 Department of Diagnostics and Public Health, University of Verona, Strada le Grazie 8, 37100 Verona, Italy; 2 Azienda Ospedaliera Universitaria Integrata Verona, Verona, Italy

**Keywords:** Academic failure, Determinants, Drop-out, Education policy, Nursing students, Attrition, Retention

## Abstract

**Background:**

Over the past few years, the phenomenon of “nursing student attrition” has been unevenly studied. Investigators often focused on independent predictors as age, family obligations, final grade of high school, demanding physical and mental workload and others. Specifically, just a few studies applied qualitative methods to better comprehend the very needs of first year students enrolled in a bachelor’s degree in nursing sciences (BSN), to sustain their learning process and define effective strategies to reduce student drop-out.

**Methods:**

We conducted a qualitative descriptive study. Thirty-one nursing students at Verona University were interviewed using a semi-structured guide. Data analysis was performed according to a descriptive approach by Sandelowski & Barroso (2000).

**Results:**

A total number of 31 students were interviewed. The most recurrent themes regarding the reasons behind BSN drop-out were: understanding that they were not suited to be nurses, perception of missing/lack of psychological, physical and practical resources needed to successfully cope with both nursing school and the nursing profession, inconsistencies between the image of the profession and the reality of the job, feelings of disappointment for the experiences of internship, perceived lack of support from the clinical teacher while going through difficult experiences.

**Conclusions:**

We can consider a part of these drop-out decisions normal, even physiological when students come to realise that they are not suited for the nursing profession. However, it’s important to guide nursing students with adequate counselling in order to give them the essential tools to cope with the training and the future as health professionals.

## Background

Nurses represent a key element in healthcare systems thanks to their involvement in the different areas of disease prevention, delivery of primary health care, treatment and rehabilitation. For this reason, nursing education has started to be delivered by academic institutions, therefore students are required to attend the Bachelor of Science in Nursing in order to obtain their degree.

Globally, every given year thousands of students decide to begin their pathway in higher education enrolling in a bachelor’s degree with a major in Nursing, however not all the students who begin are then able to successfully complete the program and gain their bachelor’s degree [1, 2].

Many authors have highlighted the global shortage of healthcare professionals [1, 3, 4], specifically the World Health Organization has foreseen a shortage of 18 million healthcare professionals by 2030, whereby half of the need are nurses [5]. Such global shortage is the result of the increasing demand for population health services, due mainly to two phenomena: the increasing number of people living with chronic conditions and ageing population, coupled with a shrinking workforce, with more nurses retiring from the profession and others leaving the workforce as result of challenging work environments [5–7].

Therefore, it becomes of the utmost importance to understand in a more focused way the main reasons behind the dropout phenomenon in nursing students, in order to be able to favour student retention and prevent further depletion affecting the health force. For this reason, the international literature has recently started to focus the attention on the prevalence of the phenomenon of attrition in nursing students and some efforts have been made to define risk factors in relation to attrition as well as potential preventive strategies [8].

University attrition constitutes a challenge under many different aspects: 1) the economic burden suffered by families due to the fact that their investment won’t translate into a valuable qualification [9]; 2) the capital invested in drop-out students, and therefore lost, by universities [10]; 3) the social costs related to the failed retention of health professionals which won’t access the market [11, 12], translating in a general decrease of growth and innovation for the country as well as a potential decrease of the quality of care provided by the healthcare system due to the shortage of professionals.

The matter of university attrition constitutes a relevant topic for Italian nursing faculties. The European Union (EU) in 2015 published a report [13] highlighting a 33% attrition rate of Italian students attending the Bachelor of Science in Nursing, whereas in 2013, the Italian National Agency for the Evaluation of Universities and Research Institutes had reported an even higher 38,7% drop out rate. As regarding the data of single institutions, Palese [14] in 2009 reported a retention failure of 19–20%, while Dante highlighted a 35–37% in 2011 [15].

The wide variability in drop-out rates found by researchers in Italy is reflected also internationally. In England, for example, the drop-out range varies from 25 to 40% depending on the study [16]. Canada, United States and Australia present variations in drop-out going from 10 to 50% [4, 17]. The reason behind such different numbers can be found in the lack of consensus regarding the definition of “attrition” or “drop-out”. It would be crucial to clearly define the phenomenon in order to have more reliable data. A shared definition would enable researchers to establish what percentage of student drop-out may be generally expected and what threshold may instead constitute the reflection of an internal issue of the single academic centre. The determinants of attrition among nursing students are many and complex to assess and the diverse connotations that the term “drop-out” often assume at the international level make comparisons between the Italian academic context and other international contexts challenging.

In 2015, the European Commission’s Directorate General for Education and Culture proposed “to increase the number of graduates from tertiary education to at least 40% of 30- 34 year old by 2020” in order to lower the drop-out rate of universities. Although Europe as a whole has achieved this objective, Italian universities haven’t been able yet to meet this target [18].

Moreover, the matters of university retention and attrition have been known and debated by the academic literature since the ‘60s [17], but the study of these dynamics has been fragmentary and uneven. Most of the recent studies focused on single predicting factors of attrition such as age, family obligations, high school proficiency, demanding physical and mental workload and others. Other authors investigated retention, which represents the other side of the same coin, although not always capable to inform researchers regarding the reasons for drop-out. Furthermore, very few studies have used qualitative methods with a wide range of participants, which would allow a deeper comprehension of the phenomenon from the students’ perspective.

Palese defined in 2009 two main reasons of attrition for nursing students. The first can be considered “physiological”, because it refers to the natural phenomenon of “awakening”: students which start their academic pathway and along the way come to realise that they are not suited for the nursing profession [14]. This consciousness, which leads some students to drop out from their BSN, is not only positive, but also desirable, because it is the best way to train future professionals highly committed to their profession [19]. However, there is also a “pathological” reason leading students to drop out from nursing school and it refers to those students who are instead suited for the profession but decide to not continue in their nursing program for other reasons. Examples of these reasons may be related to the student’s scarce proficiency or to other personal issues such a family obligations or difficult conciliation with work schedules [14]. This decision translates into the loss of valuable professionals for the healthcare system, therefore it’s important to define which abandonments should be avoided and how to prevent them from happening.

Quantitative research is effective in highlighting drop-out rates, it is however not suited to thoroughly investigate the causes behind nursing students’ attrition. Therefore, we decided to conduct a qualitative study in order to explore the motivations behind drop-out, listening to the direct experiences of the nursing students.

### Aim

The primary aim of this study is to investigate the reasons behind nursing students’ attrition at the University of Verona. The research question, which guided the study was: what are the reasons behind the nursing student’s attrition phenomenon?

## Methods

### Design

A qualitative descriptive study was conducted.

### Setting

The study took place at the faculty of Nursing of Verona University, which offers a three-year bachelor’s degree in Nursing (160 ECTS, including 60 ECTS of clinical training). During the first year, students have courses in anatomy, physiology, chemistry, biology, public health and fundamentals of nursing practice. During the second and the third year of the program, the courses are more focused on gaining clinical and pharmacological knowledge with respect to the main clinical conditions and care management in the areas of medical and surgical practice, evidence-based practice, ethics, and organizational administration. During the 3 years, students have to complete eight clinical placement experiences (five to 6 weeks for each experience) with two in the first year and three in the second and third years, respectively. These experiences include hospital-based clinicals (medical, surgical, geriatric and intensive care units), home care, community services, and an experience in a Skilled Nursing Facility. During their clinical placements, students are supported by different tutor roles, including academic clinical teachers and preceptors working in the clinical settings.

### Sample

Convenience sampling was adopted. All the students which were initially enrolled in their first year of BSN in 2018/2019 and decided to drop-out from their undergraduate degree were asked to participate to the study. Students who presented formal request of withdrawal from the course, and those who did not formally abandon the program but stopped to attend mandatory clinical activities, were considered as they were dropping out from the BSN. As qualitative method driven the study a sample size determination was not a prior defined, instead a relies on the richness of data collected until data saturation [20], were considered.

### Data collection

Students were contacted by e-mail in order to investigate their willingness to participate to the study.

Students who replied with a positive answer were contacted to set up a face to face semi-structured interview, performed by a trained investigator and recorded with the aid of a digital audio recorder.

The interview guide consisted of 7 open-questions: “When did you leave the program?”; “What is your main reason for leaving the program?”; “Are there any other reasons that led you to leave the program?”; “Did you expect something different from the program or from your internship/placement?”; “Did you like the nursing profession?”; “Is there something that maybe would have helped you to remain in the program?”

Every question was open to let the interviewed person talk about his/her experience as he/she deemed appropriate. At the end of the interview all students were asked to provide some demographic data such as: age, gender, place of residence, admission test score and drop out date/period. To ensure a high degree of consistency, all interviews were conducted by the same researcher.

### Data analysis

The same researcher (AC) firstly listened to the recorded interviews several times and then transcribed them verbatim after each meeting. Data were subsequently analysed using a descriptive qualitative approach [21].

Each interview was carefully and repeatedly read in order to obtain a general sense of the content. Two members of the team independently identified and marked the most significant words and phrases (units of meaning), using an open coding approach and;

reducing each significant element to a descriptive label (code. After this phase, the team all together grouped the codes emerged into categories according to their similarities. As last step

through the examination of each category, its meaning and how often it occurred in the data, homogeneous categories were merged to form five themes The transcripts were anonymised and representative quotations from the transcribed text were used to support findings.

### Rigour and trustworthiness

The rigour and the trustworthiness of the study was pursued using different strategies. The PI, a registered nurse, collected all data; she had not previously met the participants and she took notes regarding personal feelings, biases and insights immediately after each interviewThe principle of saturation was applied within convenience sampling to achieve the sample sizes of 31 research interview participants. The analyses of all data were done independently by two researchers (AC and FC), both nurse educators with previous experience in qualitative research. Disagreements were resolved by discussion. The participant’s own words within each of the marked units of meaning were analysed to grasp the meaning for each participant. Simultaneous to this, the researchers practiced self-reflexivity via their own research journals to name, analyse and reduce bias. The reflexive journals allowed the researchers to become aware of their own preconceptions and their theoretical and ideological assumptions to minimize the influence of preconceived perspectives. Moreover, the journal was used by each researcher to record also methodological notes, providing a detailed description of the research methods anddocumenting all the steps and critical points in the research trajectory [21, 22]. To maximize data accuracy, member checks were carried out with all participants after all the interviews, and participants’ words were used to support categories’ description.

## Results

### Sample characteristics

On a total of 274 first-year undergraduate nursing students at the University of Verona attending the academic year 2018/2019, 42 students decided to drop-out from the bachelor’s degree in nursing. Out of the 31 students which decided to drop-out, 5 males and 26 females accepted to be interviewed. The mean age of the interviewed students was 21 years old (range 19–26 years old), the same as the average of the students who decided to continue the undergraduate nursing program. Among the students who decided to leave the BSN of the University of Verona, 12 of them did not have official residence in Verona’s County. Among these 31 students, 1 student decided to withdraw from the program right after enrolment, before the official beginning of the academic activities. Five other students left the program because they had become eligible to attend another faculty, which was the one they preferred. Other 12 students left during the first semester of theory lessons, 15 students left during clinical training, 3 students after the first days of clinical placement, 8 at the end of their first clinical placement while the last two at the end of their second clinical placement. The process of analysing interviews resulted in five themes, which are synthesized in Table 1 and described in the following paragraphs.

**Table 1:** Motivations concurring to the final decision to drop-out from the BSN (from the most to the least reported by students)

Themes	Categories
Understanding of not being suited to be a nurse	Feeling inadequate for the nursing profession
	Having the desire to try another profession
Perception of lacking the psychological, physical, and practical resources needed to successfully cope with both nursing school and the nursing profession	Being unable to manage emotions
	To struggle with academic demands
	Organization skills
	Commitment (self-awareness)
	Not Effectively cope with stress
	Concerns due to personal/family circumstances
	To be suffering financial problems
	To have health issues
	To have language issues
Incongruity between one ideal or perfect idea of the nursing profession and the real impact with it during the clinical placement	To not find in the real world the human dimension of the job anticipated by the theory lessons
	To experience teamwork without then finding it in every clinical context
	To not associate caring competence to the nurse
To perceive lack of support from the clinical teacher	To wish for a closer relationship and more support from the clinical teacher
	To wish for more interest from the clinical teacher’s point of view
	Expect help from the clinical teacher
	Find it hard to open up with people about your own issues
	To not establish a trust relationship with your clinical teacher
To not be satisfied with the overall experience of placement	Wish to have some time dedicated to reflection
	To wish for more mentoring/mentorship
	To perceive as negative the simultaneous presence of multiple students in the same unit
	To wish for more opportunities to experience the job while in placement
	To have the opportunity to observe a good role models during training activities
	To negatively handle the relationship with the team

### Understanding of not being suited to be a nurse

One of the reasons pushing students to drop-out from the nursing undergraduate is the realisation of not being suited to be a nurse. Some students begin to question their decision during the first semester of theory, however the turning point for the decision of leaving the BSN is more often represented by the simulation labs and, especially, by the first clinical placement: *“Initially I liked it a lot, I was really invested and convinced that nursing was going to be my future career, but then I realised that it was not for me, I didn’t feel suited for this job. I do not know why, but I did not feel in the right place. I understood that I was not in the right place also because of thsimulation labs. I realised that, thinking about the training experience of the day, in the evening I was not satisfied about how I was living the training experience, not because of the training itself but because of my perception of it.”*

During this first placement students can in fact be “*socialized to the job*”. They can start to discover the professional identity of the nurse, the related sphere of action and therefore begin to understand if it may be the right pathway for them. Indeed, students tend to drop out when they begin to experience real contact with clinical situations involving real patients and their suffering, as this student pointed out. *“I understood that nursing wasn’t the right career pathway for me. I realised that in this profession there is a lot of contact with the patients and at the beginning I didn’t know this, so I realised that I didn’t feel suited for this.”*

Moreover, students reported to feel inadequate in performing caring actions, which involving situations of intimacy, such as bahing and personal hygiene.

*“I think that [being a nurse] it’s a good thing but you have to be suited for it. I don’t feel comfortable in helping others with their personal hygiene, I’m not able to cope with this kind of situation.”*

Some students declare that they decided to start their bachelor’s in nursing because they did not successfully pass the entrance test for the school of medicine, but their desire to become a physician remained their priority and pushed them to leave the BSN, as reported by this student:

*“Then [when I started to attend the BSN] I saw that my desire to attend medicine was still strong. I would have seen myself more as a doctor rather than a nurse, I will become a doctor, this is my plan A, it has been my dream since I was a child.”*

### Perception of lacking the psychological, physical and practical resources needed to successfully cope with both nursing school and the nursing profession

Human beings must constantly learn to develop new skills to tackle the challenges brought by daily life. These skills may be innate characteristics of the individual or they may be built in response to stimuli and stressful situations. Sometimes students realise that they lack the right resources to successfully cope with challenging situations and they decide to drop-out from the BSN.

*“I suffer from anxiety, and I cannot think of leaving my hometown. [At the beginning] I started to drive to Verona every day to attend the theory lessons, but when the program became more intense and time-consuming, I did not want to come anymore, and I quit. I think that I have decided to drop out when I found out that I would have had to start my first clinical placement soon and therefore I would have had to stay in Verona for the night shifts. The idea of being in Verona at night was extremely frightening for me and I decided to stop my university career in nursing.”*

Some students may struggle with managing their emotions. Some of them reported in the interviews their struggles to cope with their emotions in delicate situations, like when they see a patient suffering:

*“It was psychologically very hard; you cannot be fragile if you must care for all those people who are sick and suffering. For this reason, I preferred to leave the course.”*

The transitional phase from high school to university is challenging and, differently from high school, the student needs to develop adequate organization skills to keep up with the study, ending up with multiple different arguments to study right before the exam session: *“I tried to study to pass all the exams during the first semester, but this strategy was a failure because I was studying a bit of every subject and that led me nowhere. There are many different subjects and it’s difficult to organize the study of so many different arguments at the same time.”*

The perception of having the psychological, physical and practical resources needed to successfully cope with both nursing school and the nursing profession is an essential component for student retention; however, it clearly emerges from the interviews that motivation is the key factor to successfully continue this academic pathway.

*“I wasn’t motivated enough to continue with nursing. I’ve observed my fellow colleagues during the placement, and they were much more motivated than me. After the shift they would review the clinical cases explained while we were at the hospital, but I didn’t have the motivation to do this and I thought that maybe I need to find something that gives me the same commitment that my colleagues have for nursing.”*

### Incongruity between the ideal of the nursing profession and the real impact with the profession during the clinical placement

The “socialization to the profession” is not immediate, it is preceded by a time where students build up their idea about the job and they create their own expectations.

During their first clinical placement students’ expectations meet the job reality. This on one hand allows students to appreciate characteristics and capture details about their future profession that they could only imagine before training. However, this first contact may reveal elements of dissonance between what the students were expecting and the reality of the nursing profession. Two students shared with us this feeling:

*“In class we had talked a lot about caring and the human side of this profession and I was wondering if in the real world I would have had possibility to dedicate part of my time as nurse to this. I tried to give my interpretation of the profession. I noticed that as a student I had more time to dedicate to my patients, I had more time to spend in their room also because I was assigned to the healthcare assistant, or when I was doing some activities, or while my nurse supervisor was administering the therapy, I had more time to talk with patients. I noticed that all the nurse supervisors I had were always in a hurry. They were all always busy administering medications, running from a room to the other, I didn’t expect something from them so mechanical and schematic, I was hoping for something more focused on human contact with patients.”*

While some students reported the mismatch between the ideal of the nursing profession clashing with the impact of the reality of the profession given by the clinical placement, others had a mistaken idea of the nursing profession. Indeed, they didn’t associate caring and nursing assistance to the role of the nurse. Moreover, they didn’t consider that nurses have not only to entertain relations with patients but also to work in team with other professionals.

The students words suggested that the socialization to the nursing profession is not immediate and it is often influenced by the students previous idea about the job.

### To perceive lack of support from the clinical teacher

The student may encounter many challenges during his/her learning process, and for this reason he/she would like to have a close relationship with his/her clinical teacher.

Some students perceived distance or indifference from their clinical teacher at the time when, in their opinion, they needed him/her the most and the lack of support translated in drop-out from the course:*“I said to the teacher that I couldn’t come to the hospital unit for a couple of days because I didn’t feel well, I didn’t know if I wanted to continue with the BSN or not and she just kept answering me ‘it’s all right, it’s all right’. I know for a fact that I need to be supported closely, I give a lot, but I also need to receive back from people, to feel that I can count on them. Maybe if I’d have had a teacher more invested in me, things might have been different.”*

Other students didn’t share the decision of leaving the BSN with their clinical teacher because they didn’t feel they had a relationship of trust with them. In some cases, students declared that their discomfort wasn’t related to their lack of relationship with the teacher, but it was more due to their own difficulty to admit that they were struggling:

*“It’s not always easy to open up to people, tell them that you are having a hard time, you should spend time with them more often [to build the courage necessary to talk about these issues].”*

### To not be satisfied with the overall experience of clinical placement

Often the clinical placement is a challenging moment for the student. Expectations about the nursing profession can clash with a busy and understaffed reality and the complex combination of lack of workforce and high workload may lead the student to feel like an additional burden for many clinical settings.

Students may be slower in the execution of clinical or caring procedures due to their lack of practice and their learning still in progress. Longer execution times may become a source of frustration for both nurse supervisors and students:

*“If I stayed in a room longer than my supervisor, she would reproach me because we had to go to make a bed in the other room. I needed more time to practice how to make the bed, but I was scolded because I wasn’t ready to do something else soon enough for the nurse’s schedule.”*

The student may negatively experience the relationship with the team, both because he/she may feel that the nurses of the shift do not dedicate enough time to him/her or because the nurses do not establish educational relationships with the student. The student would like to follow his/her nurse supervisor or clinical teacher during the shifts because they represent the role model for the profession that he/she would like to pursue in the future. For this reason, students find discomforting to be assigned to follow support staff like healthcare assistants:

*“My nurse supervisor had a part-time contract; therefore, I barely saw her during the placement. I was always with the healthcare assistant. I hated it because it didn’t give me the opportunity to see all the aspects of the job I would have had to do in the future. I didn’t pretend to not work at all with the support staff, but I would have liked more balance. I would have liked to understand practically what I was going to do as a nurse. If I wanted to be a healthcare assistant, I would have chosen that pathway and not a nursing degree.”*

Students appreciate to experience relations with different professional figures and to work in team, nevertheless they would like to spend more time with their nurse supervisor in order to fully understand the reality of the nurse profession. Furthermore, the simultaneous presence of large groups of students in the hospital unit is considered by some students an obstacle for the learning process:

*“Sometimes, it has happened that the nurse supervisor, after having seen many students in the unit for the same shift, started to assign tasks to every student at the beginning of the shift. His focus was to give to everyone something to do, but we were more interested to follow him and understand why he was doing something in a certain way, we wanted to learn the process and the motivations behind every move.”*

## Discussion

At a certain point of the life pathway, every person must decide the work career more suitable for him/her, and this is a key moment for the life of every individual. The students that, after high school, decide to further their studies with an academic degree, have the same kind of dilemma: the choice of the right faculty is a key component to start to build a bright future.

Although most of the students arrive to the decision of which bachelor to pursue after careful reflections and considerations, some of them begin to doubt the validity of their decision early after the beginning of the academic year [9, 23]. From our study it emerges that students begin to question their decision during the first semester of theory, however the turning point for the decision of leaving the BSN is more often represented by the first clinical placement. As a matter of fact, during the first training experience nursing students may start to discover their professional identity, as reported by the present study. Bowden [24] and Loberto [25] highlighted that students tend to drop out when they begin to experience real contact with clinical situations involving real patients and their suffering.

Moreover This study raised an important element to take into account considering students’ drop- out, It appears that students may struggle when they experience the most intimate sphere of caring, assistance with personal hygiene as Dal Santo and colleagues found in their study on first year nursing students experiencing their first clinical placement [26]..

The perception of having the psychological, physical and practical resources needed to successfully cope with both nursing school and the nursing profession seems to result an essential component for student retention. This component is not limited to the element of resilience, already showed by Crombie [9] and Knight [27], on the contrary, it includes also life skills as the ability to manage emotions (like anxiety), self-awareness, organizational skills and the commitment to complete the program. These abilities enable students to deal with complex situations not only at the university or at work, but also in their personal life. On the other hand, the capability of keeping up with the academic demands and therefore pass the exams is a specific skill essential for successful academic retention. Previous investigators [24, 28] raised concerns related to the ability of nursing students to cope with exam stress. From the results of this study it appears that the issue is not really related to the ability to cope with excessive stress for the exams, but instead related to successfully deal with both the academic and the clinical workload, and some students seem to lack the organization skills needed.

Moreover, one of the reasons pushing students to drop out from the program,, is posed by the mismatch between the ideal of the nursing profession clashing with the clinical placement impact of the reality of the profession. Prymachuk [29] and Wells [30] talk about “disillusionment”, indicating that students who left were more likely than students who remained enrolled to report a discrepancy between what they expected and the realities of nursing education. However, these authors didn’t investigate deeper the roots of this disillusionment. From our research it appears that one of the causes behind this phenomenon may lay with the fact that students do not always find in their placement experience the human dimension of the job, as instead expected and anticipated by the theory lessons. From the theory lessons students learn about the importance of the relational aspect of caring and to look at the patient with a holistic approach. Despite this, they may encounter in practice another reality, where sometimes the hectic pace of activities force nurses to prioritize the clinical aspect of their job over the relational one.

Another of the reasons reported by the literature as cause for attrition was constituted by distress situations with the mentor. Issues of negativity and prejudice in mentors were cited as being frequent and commonplace, putting a heavy burden on the shoulders of students. It has been shown that experiences in clinical practice have the greatest influence on students wanting to stay on the program [14, 17, 19, 27, 29, 31]. In our study, the mentorship aspect revealed many different components. On one hand, some students perceived distance or indifference from their clinical teacher at the time when, in their opinion, they needed him/her the most and the lack of support translated in drop-out from the course. Moreover, the fast pace typical of hospital units has been showed as challenging for the students, due to the short time that nurses could dedicate to them and to the patients, and due to the nurses’ high expectations regarding the student’s practical skills, not in line with the student’s level of ability at that time. On the other hand, students declared that they desired to shadow more their role models and to spend a minor amount of time with support figures like nurse assistants.

Lastly, another issue specifically highlighted by this cohort regards the difficulties of some first year nursing students that have to learn to toilet, shower, and dress patients as one of the first and foremost activities during their first year, even if they would instead prefer to have the opportunity to shadow their nurse supervisor and see every aspect of the job from the beginning [26]. To address this issue, it may be useful to take a different approach, for example to lower about the number of hours that students spend with nurse assistants and to teach the actions of caring related to personal hygiene applied to complex clinical situations, such as critical care patients.

According to the results of this study it is possible to affirm that for some of the students, which became aware of not being suited to be a nurse, attrition is something unavoidable. The phenomenon of attrition bears a negative connotation related to the negative judgement of failure to complete the degree. On the contrary, our study shows a different perspective. Indeed, it is possible to notice how the interviewed students do not use negative words as “error” or “mistake”, but rather expressions denoting understanding – “I understood”, “I realised that I am not suited”, “I do not feel it like something for me”-. These students feel some difficulties, they ask themselves what to do and even if they struggle with these doubts, they finally decide, with peace of mind, to leave a program which they do not perceive as the right career pathway. It is important to accompany students during this process, and to not try to stop this decision, but to make sure they follow their aspirations and skills. Such approach is confirmed by the view of Urwin et al. [19] who propose that some attrition is inevitable and even desirable in order to maintain standards within the profession.

The present study has some limitations. Firstly, it described reasons of drop-out from the perspectives of students attending only one Italian Bachelor’s Degree program in Nursing and deciding to leave only within the first year of the course. Another limitation is that only the students who actually stopped but not the students who considered stopping but continued, were questioned.

Further exploration in other nursing academic settings is needed in order to give a deep understanding of the nursing student attrition. Additional research exploring the effectiveness of strategies to improve nursing students’ intention to stay could provide valuable additional knowledge for nursing education.

## Conclusions and implications for practice

It is relevant for educators having insight on the perspectives of nursing students involved in the phenomenon of attrition. This knowledge leads to develop some strategies that may prevent this phenomenon. .

Some useful initiatives should be considered in order to at least reduce the evitable component of nursing student attrition: the first may be constituted by the creation of open days/weeks events targeting high school students, where students have the chance to attend nursing classes for a number of days and better understand what the main components of nursing undergraduate programs are. It is important to highlight that these approaches may disproportionately emphasise the advantages of the program and career, potentially setting unrealistic expectations. On the other hand, detailed information about programmes, being a nursing student, and the role of nurses in the health workforce would enable candidates to self-select against the programme requirements [32] and become a winning strategy to favour retention. Another idea to better prepare candidates has recently tried by the Scottish health department, which developed a repository of digital resources based on the theme of ‘Extra-ordinary Every Day’ to encourage potential nurses and midwives to reflect on the values, attitudes and capabilities they may need to succeed in these professions in the twenty-first century [7].

The second suggested intervention is targeted at those students lacking the psychological the psychological, physical or practical resources needed to successfully cope with both nursing school and the nursing profession. In this regard, there may be multiple strategies available to support struggling students. It is vital in this case to support the student, and a trained nurse educator may offer counselling, with the final aim to help the student to develop these professional skills and achieve his full potential. The teachers of the BSN could have a crucial role in identifying those students who seem to be struggling and support them using a tailored approach such as one-to-one discussions and debriefings [33, 34]. Peer mentoring is another very useful resource highlighted by the literature as effective support for nursing students. Peer leaders act as a liaison to multiple resources, opportunities, and appropriate personnel, as well as sharing learning strategies and provide the students with a safe space to share their experiences [35, 36].

Furthermore, the creation of summer schools for future first year students might help those students with lower admission scores to prevent struggles further down the academic pathway. The aim of these initiatives is to prepare candidates who had been out of education for a while, or to provide intensive training to bring candidates up to the entrance standard [7].

Moreover, for those students who decide to leave as a result of difficulties and issues related to their clinical placement. In this instance, tutorship may be at the heart of both the problem and the solution. The student should be put in condition to absorb and learn from each clinical placement, regardless of the setting, and the clinical teacher bears the responsibility of ensuring that the student is supported through this training process, for example making sure that the student is assigned to positive role models. Despite this, it is not recommended to try to sweeten and embellish the reality of the clinical settings, a future registered nurse should be trained to cope with working environments that sometimes may be challenging. Also in this case, supportive mentorships and the intervention of peer leaders may be the winning strategy to prevent avoidable dropouts. Effective mentorship may be limited due to time constraints and the high number of students compared to the limited number of tutors. However, mentors may ask the support of the ward nurses which had followed the student over the last (or previous) clinical placement and that had established a successful relation with the student.

## Data Availability

The dataset used in the current study can be made available by the corresponding author upon reasonable request.

## Abbrevations

ECTS: European credit transfer and accumulation system
BSN: Bachelor of science in nursing

## References

[1] Aluttis C, Bishaw T, Frank MW (2014). The workforce for health in a globalized context--global shortages and international migration. Glob Health Action.

[2] Harris RC, Rosenberg L, Grace O'Rourke ME (2014). Addressing the challenges of nursing student attrition. J Nurs Educ.

[3] Buchan J, O'May F, Dussault G (2013). Nursing workforce policy and the economic crisis: a global overview. J Nurs Scholarship.

[4] Hamshire C, Jack K, Forsyth R, Langan AM, Harris WE (2019). The wicked problem of healthcare student attrition. Nurs Inq.

[5] WHO (2020). Global strategy on human resources for health: Workforce 2030.

[6] Both-Nwabuwe JMC, Dijkstra MTM, Klink A, Beersma B (2018). Maldistribution or scarcity of nurses? The devil is in the detail. J Nurs Manag.

[7] Twigg D, McCullough K (2014). Nurse retention: a review of strategies to create and enhance positive practice environments in clinical settings. Int J Nurs Stud.

[8] Bakker EJM, Verhaegh KJ, Kox J, van der Beek AJ, Boot CRL, Roelofs P, Francke AL (2019). Late dropout from nursing education: an interview study of nursing students' experiences and reasons. Nurse Educ Pract.

[9] Crombie A, Brindley J, Harris D, Marks-Maran D, Thompson TM (2013). Factors that enhance rates of completion: what makes students stay?. Nurse Educ Today.

[10] Rapporto biennale sullo stato del sistema universitario e della ricerca. Italy: National Agency for the Evaluation of Universities and Research Institutes; 2013. https://www.anvur.it/rapporto-biennale/rapporto-biennale-2013/.

[11] Beauvais AM, Stewart JG, DeNisco S, Beauvais JE (2014). Factors related to academic success among nursing students: a descriptive correlational research study. Nurse Educ Today.

[12] Humphries N, Crowe S, Brugha R (2018). Failing to retain a new generation of doctors: qualitative insights from a high-income country. BMC Health Serv Res.

[13] Kottmann A, Betts A, Bitusikova A, Boudard E, Boyadjieva P, Brown M, Carlhed C, Cremonini L, Decataldo A, Doolan K (2015). Dropout and completion in higher education in Europe. Annex 2: short country reports.

[14] Palese A, Dante A, Valoppi G, Sandri G (2009). Verso il monitoraggio dell’efficienza universitaria. Fattori di rischio di abbandono e di insuccesso accademico nei Corsi di Laurea in Infermieristica. Med Chirurgia.

[15] Dante A, Valoppi G, Saiani L, Palese A (2011). Factors associated with nursing students' academic success or failure: a retrospective Italian multicenter study. Nurse Educ Today.

[16] Why as many as one in four nursing students could be dropping out of their degrees [https://rcni.com/nursing-standard/newsroom/news/why-many-one-four-nursing-students-could-be-dropping-out-of-their-degrees-137661].

[17] Merkley BJ (2015). Practice: student nurse attrition: a half century of research. J Nurs Educ Pract.

[18] Eurostat: The EU has reached its tertiary education target. European Union; 2020. https://ec.europa.eu/eurostat/documents/2995521/10749941/3-22042020-BP-EN.pdf/04c88d0b-17af-cf7e-7e78-331a67f3fcd5.

[19] Urwin S, Stanley R, Jones M, Gallagher A, Wainwright P, Perkins A (2010). Understanding student nurse attrition: learning from the literature. Nurse Educ Today.

[20] Polit DF, Beck CT (2017). Nursing research: generating and assessing evidence for nursing practice, tenth edition edn.

[21] Sandelowski M, Barroso J (2002). Reading qualitative studies. Int J Qual Methods.

[22] Graneheim UH, Lundman B (2004). Qualitative content analysis in nursing research: concepts, procedures and measures to achieve trustworthiness. Nurse Educ Today.

[23] Maher BM, Hynes H, Sweeney C, Khashan AS, O'Rourke M, Doran K, Harris A, Flynn SO (2013). Medical school attrition-beyond the statistics a ten year retrospective study. BMC Med Educ.

[24] Bowden J (2008). Why do nursing students who consider leaving stay on their courses?. Nurse researcher.

[25] Loberto F, Terzoni S, Destrebecq A (2012). Indagine trasversale sull’abbandono del Corso di Laurea in Infermieristica presso l’Università degli Studi di Milano. L'infermiere.

[26] Dal Santo L, Ambrosi E, Maragna M, Marognolli O, Canzan F (2020). Nursing students' emotions evoked by the first contact with patient's body: a qualitative study. Nurse Educ Today.

[27] Knight J, Corbett A, Smith C, Watkins B, Hardy R, Jones G (2012). "what made me stay?" a review of the reasons student nurses enrolled in a bachelor of nursing programme completed their studies: a descriptive phenomenological study. Nurse Educ Today.

[28] Dante A, Ferrão S, Jarosova D, Lancia L, Nascimento C, Notara V, Pokorna A, Rybarova L, Skela-Savič B, Palese A (2016). Nursing student profiles and occurrence of early academic failure: findings from an explorative European study. Nurse Educ Today.

[29] Pryjmachuk S, Easton K, Littlewood A (2009). Nurse education: factors associated with attrition. J Adv Nurs.

[30] Wells MI (2003). An epidemiologic approach to addressing student attrition in nursing programs. J Profess Nurs.

[31] Lancia L, Petrucci C, Giorgi F, Dante A, Cifone MG (2013). Academic success or failure in nursing students: results of a retrospective observational study. Nurse Educ Today.

[32] Rodgers S, Stenhouse R, McCreaddie M, Small P (2013). Recruitment, selection and retention of nursing and midwifery students in Scottish universities. Nurse Educ Today.

[33] Raines D (2019). Effective mentoring is key to enhancing practice and developing the next generation of nurses. Evid Based Nurs.

[34] Lee J, Lee H, Kim S, Choi M, Ko IS, Bae J, Kim SH (2020). Debriefing methods and learning outcomes in simulation nursing education: a systematic review and meta-analysis. Nurse Educ Today.

[35] Miller H, Bosselait L, Venturato L, Irion K, Schmidt N, DiGeronimo J, Pritchard T (2019). Benefits of peer mentoring in Prelicensure nursing education: a dual perspective. Nurse Educ.

[36] Pålsson Y, Mårtensson G, Swenne CL, Ädel E, Engström M (2017). A peer learning intervention for nursing students in clinical practice education: a quasi-experimental study. Nurse Educ Today.

